# Development of a High-Throughput Molecular Imaging-Based Orthotopic Hepatocellular Carcinoma Model

**DOI:** 10.7759/cureus.281

**Published:** 2015-06-27

**Authors:** Gloria L Hwang, Maurice A van den Bosch, Young I Kim, Regina Katzenberg, Juergen K Willmann, Ramasamy Paulmurugan, Sanjiv S Gambhir, Lawrence Hofmann

**Affiliations:** 1 Radiology, Stanford University School of Medicine; 2 Radiology, University of Utrecht; 3 Radiology, Seoul National University College of Medicine

**Keywords:** small animal model, hepatocellular carcinoma, molecular imaging, tumor growth, bioluminescence imaging, positron emission tomography

## Abstract

We have developed a novel orthotopic rat hepatocellular (HCC) model and have assessed the ability to use bioluminescence imaging (BLI), positron emission tomography (PET), and ultrasound for early tumor detection and monitoring of disease progression.

Briefly, rat HCC cells were stably transfected with click beetle red as a reporter gene for BLI. Tumor cells were injected under direct visualization into the left or middle lobe of the liver in 37 rats. In six animals, serial PET, BLI, and ultrasound imaging were performed at 10-time points in 28 days. The remainder of the animals underwent PET imaging at 14 days. Tumor implantation was successful in 34 of 37 animals (91.9%). In the six animals that underwent serial imaging, tumor formation was first detected with BLI on Day 4 with continued increase through Day 21, and hypermetabolic activity on PET was first noted on Days 14-15 with continued increase through Day 28. PET activity was seen on Day 14 in the 28 other animals that demonstrated tumor development. Anatomic tumor formation was detected with ultrasound at Days 10-12 with continued growth through Day 28. The first metastases were detected by PET after Day 24.

We have successfully developed and validated a novel orthotopic HCC small animal model that permits longitudinal assessment of change in tumor size using molecular imaging techniques. BLI is the most sensitive imaging method for detection of early tumor formation and growth. This model permits high-throughput *in vivo* evaluation of image-guided therapies.

## Introduction

Hepatocellular carcinoma (HCC) is the fifth most common neoplasm in the world, with a worldwide incidence of more than 500,000 new cases diagnosed annually [1]. Despite a broad armamentarium of therapies aimed at treating HCC — including chemotherapeutic medications, percutaneous ablation, transarterial chemoembolization, radioembolization, surgical resection, and transplantation — long-term disease-free survival remains elusive in the vast majority of patients. First-line treatments include resection and liver transplantation [2]. Unfortunately, only 10% of HCC patients are eligible for surgical treatment [3]. Medications, such as sorafenib, offer only modest survival benefits in select patients [4]. Therefore, image-guided interventions, such as transarterial and percutaneous therapies, are the mainstays of therapy for non-surgical candidates.

Historically, research in image-guided interventions has relied on the use of large animals. This was driven by a focus on the development of mechanical medical devices. However, large animal experiments are extremely expensive and time-intensive. Moreover, if the longitudinal effects of a therapy are to be measured, multiple animals must enter the same treatment arm of the experiment, but then must be euthanized at different time points in order to have histologic correlation. This increases the number of animals needed to generate significant results. 

As disease treatment shifts from a mechanical to a molecular approach, the important feature of an appropriate animal model is not whether the organ or vessel size matches that of humans but rather whether the model mimics the disease at a molecular and cellular level. Many of the published studies investigating HCC image-guided interventions currently utilize the rabbit VX2 model [5-15]. This model enjoys the benefits of a convenient size for vascular catheterizations and percutaneous ablative therapies. However, it suffers from the shortcomings of large animal models, and it is limited in that it is created with a squamous carcinoma cell line and, hence, does not represent a true hepatocellular carcinoma.

In designing a model for image-guided molecular interventions for HCC, we sought a few key characteristics. First, the model must be cell-specific for HCC. Second, the model must be small enough for high-throughput studies and yet large enough to test intraparenchymal and intra-arterial delivery routes. The Morris hepatoma model — which uses rat HCC cells from an inbred immune-competent rat line — met both of these criteria [16-20]. Different techniques have been described to implant the hepatoma cells in the rat liver. We optimized the technique, described below, to reproducibly implant a single focus of tumor in the liver while minimizing the spillage of cells into the peritoneal cavity.

A third characteristic we desired in our model was the ability to serially assess the growth and response to therapy of the tumor without euthanizing multiple animals at different time points. Not only would this decrease the time and resources needed to complete the experiment, but it also would also allow us to better appreciate relatively small differences in response to therapy. One difficulty with traditional models that require euthanasia at different time points is that it is impossible to guarantee that the tumor in each subject is the same size at any given time point. In order to overcome this inherent variability, large numbers of animals must be studied to assess effect of therapy, and there is still a risk that the results are skewed by differences in tumor growth in the different study arms. To allow quantitative serial evaluation of the tumor, we stably transfected the tumor cells with a bioluminescence gene prior to implantation. We then assessed our ability to functionally and morphologically characterize the tumor with multiple non-invasive imaging modalities.

## Materials and methods

### Generation of McA-RH7777-CBRluc

The McA-RH7777 cell line, a Morris HCC cell line derived from the Buffalo rat, was purchased from the American Type Culture Collection (Manassas, VA) and grown in a humidified incubator at 37°C, 5% CO_2_ in Dulbecco’s Modified Eagle’s medium with 10% heat-inactivated fetal bovine serum sans antibiotics (Invitrogen, Carlsbad, CA). The cells were stably transfected with a retrovirus encoding click beetle red luciferase (CBRluc) [21]. In brief, replication-incompetent retroviruses were created by transfecting Phoenix-ecotropic cells with the plasmid pMSCVpuro (Clontech Laboratories, Inc., Mountain View, CA) carrying the gene for click beetle red luciferase under beta-actin promoter control. The resultant retroviral supernatant was used to transfect Morris hepatoma McA-RH7777 cells. Reporter-expressing cells were then enriched by selection with puromycin (8 ug/mL) (Sigma, St. Louis, MO) for a minimum of five passages.

### In vitro bioluminescence imaging (BLI)

To compare the bioluminescent signal with cell number, the concentration of log phase McA-RH7777-CBRluc cells, harvested eight passages after transduction, was determined by trypan exclusion assay using a hemocytometer. Cells were seeded in triplicate at a density of 1x10^5 cells/well into a 96-well, black wall tissue culture plate with clear bottom wells. Seven serial 1:1 dilutions of cell concentration were then performed, and the cells were incubated for four hours to allow adherence. Imaging was performed with the Xenogen In Vivo Imaging System (IVIS Spectrum; Caliper Life Sciences, Hopkinton, MA) after the addition of D-luciferin (0.15mg/mL final) (Biosynth AG, Staad, Switzerland). The charge-coupled device (CCD) camera was cooled to between –105º C and 120º C and the field of view set to 25 cm. The average radiance of uniform regions of interest at peak signal output was measured using Living Image 3.0 software (Caliper Life Sciences, Hopkinton, MA).

### Animal tumor model

**Figure 1:**
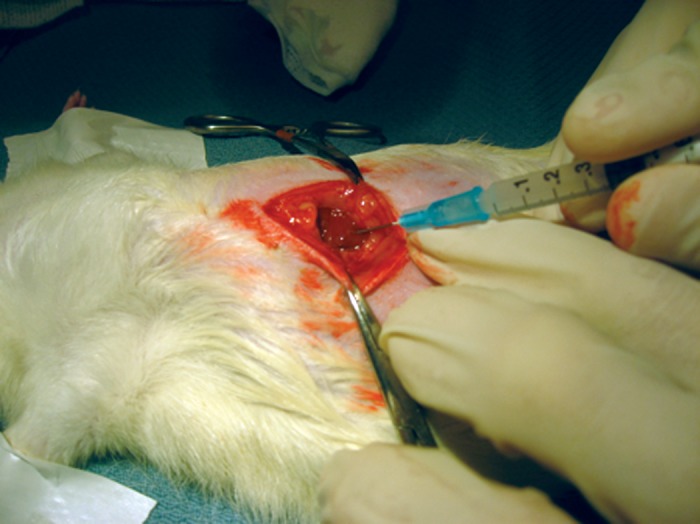
Implantation of McA-RH7777-CBRluc cells into the Buffalo rat. Through a subxiphoid vertical incision, 1.0x10^6 cells in 100µl phosphate-buffered saline was injected under direct visualization into the left lobe.

Our study protocol (Protocol #26869) was approved and monitored by the Stanford Institutional Administrative Panel on Laboratory Animal Care and performed in concordance with the ethical treatment of animals. Tumor implantation was performed in six adult male Buffalo rats (300-450 gm.) (Charles River Laboratories, Wilmington, MA). The procedure was done under anesthesia with 2% isoflurane in oxygen at 2 L/min on a heated platform. A longitudinal subxiphoid incision of approximately 2 cm was made to expose the liver. Log phase, reporter-tagged HCC cells (1.0x10^6 McA-RH7777-CBRluc in 100µL phosphate-buffered saline), used within 25 passages from the time of transduction, were injected under direct visualization through a 30-gauge needle into the left lateral lobe of the liver (Figure 1). The injection site was compressed for one minute with a cotton swab to prevent cell leakage out of the liver and/or bleeding into the abdominal cavity. The incision was closed with 4-0 chromic gut suture (Ethicon, Inc., Somerville, NJ). The animals were fed ad libitum and monitored daily.

### In vivo bioluminescence imaging

In five of the animals, BLI was performed on Day 1, Day 2, Day 4, and then every three to five days for a total of 10 time points in 28 days with the IVIS Spectrum Imaging System as previously reported [10-12]. The CCD camera was cooled to between –105º C and 120º C and the field of view set to 25 cm. Rats were anesthetized with 2% isoflurane in oxygen at 2 L/min. Ten minutes after the rats received an intraperitoneal injection of 150 mg/kg of D-luciferin in PBS, bioluminescence images were acquired with an exposure time of 1-120 sec, medium binning, 1 f/stop, with an open filter. A region of interest was drawn around the tumor, and the bioluminescence signal was quantified as photons/sec/cm^2^/steradian (p/sec/cm²/sr).

### Positron emission tomography (PET) imaging

Serial imaging of tumor growth was performed in five of the animals on Day 1, Day 2, Day 4 and then every 3-5 days for a total of 10 time points in 28 days with a dedicated small-animal PET scanner, microPET R4 (Concorde Microsystems, Knoxville, TN). After induction and maintenance of anesthesia with 2% isoflurane in oxygen at 2 L/min, the rats were injected with 1 mCi [18]F-fluorodeoxyglucose ([18]F-FDG) via the tail vein. The rats were kept under anesthesia to minimize muscle activity. After one hour, rats were imaged for 15 minutes in the prone position with a transaxial field of view of 22 cm. A region of interest (ROI) covering the tumor was drawn. The average count rate (counts/sec per pixel) within the ROI was determined and converted to tracer activity (mCi/mL) using a standard calibration constant. Assuming a tissue density of 1 g/mL, tracer activity (mCi/g) was then divided by the injected dose (mCi) to obtain a tissue-specific uptake index (percent injected dose per gram of tissue, %ID/g). Another ROI was obtained similarly in the right lobe of the liver, and tissue uptake (%ID/g) was obtained similarly to acquire liver background signal.   

Animals underwent imaging by multiple modalities on the same day under a single period of anesthetization.

### High-frequency ultrasound imaging

On Day 1, Day 2, Day 4, and every 3-5 days thereafter for a total of 10 time points in 28 days post-implantation, high-frequency small animal ultrasound (US) examination of the liver was performed in five of the animals with a dedicated small-animal high-resolution imaging unit (Vevo 770; VisualSonics, Toronto, Canada). During imaging, rats remained anesthetized with 2% isoflurane in oxygen at 2 L/min on a heated stage. Real-time imaging was performed with a 40-MHz high-frequency linear transducer. Two-dimensional B-mode images were acquired in two directions (transversal and sagittal) with manual optimization of the gain, and the maximum tumor diameter measured in millimeters was measured in millimeters using electronic calipers. Images were recorded digitally and analyzed offline using micro-US imaging software (VEVO 770; VisualSonics, Toronto, Canada).

### Euthanasia

**Figure 2:**
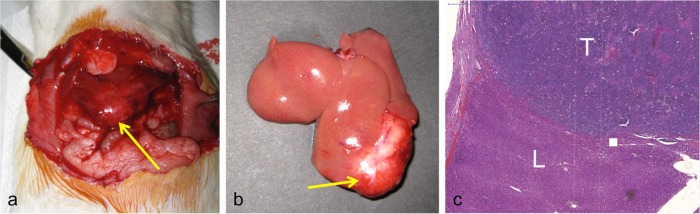
Representative gross and histopathologic specimen of Morris HCC. 5x view reveals a sharp demarcation between the implanted tumor (upper field) and the normal liver (lower field), with evidence of hypervascularity within the tumor. 20x view demonstrates that compared with normal liver (a), the HCC (b) demonstrates poorly differentiated cells, with markedly increased mitotic activity and hyperchromatism. (c) Grossly, the tumor (arrow) is pale and well-circumscribed and extends to the liver capsule.

After final imaging on Day 28, the animals were euthanized by carbon dioxide inhalation. A midline laparotomy was performed and the liver and peritoneal cavity were inspected prior to explantation of the liver. Gross pathology and hematoxylin-eosin staining from a representative liver specimen are shown in Figure 2.

### Reproducibility of cell implantation

A subsequent 31 animals underwent implantation of stably transfected McA-RH7777 cells using methods as described above. After the initial series of six animals, the decision was made to implant tumors in the middle lobe, as we found that decreased the likelihood of the tumor adhering to the abdominal wall. All animals were imaged by PET at Day 14. The three animals, which did not have tumor detectable by PET on Day 14, were euthanized on Day 14 for evaluation. The other 28 animals underwent secondary interventions and thus were not included in the longitudinal follow-up.

## Results

### McA-RH7777 cells demonstrate stable expression of bioluminescence genes

**Figure 3:**
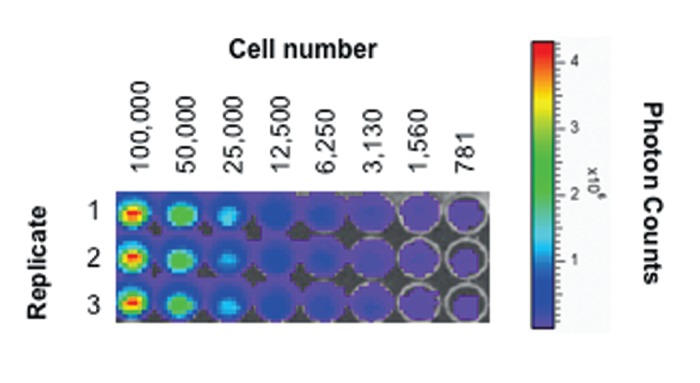
Relationship between cell concentration and bioluminescence signal in vitro. Image from 96-well plate obtained with IVIS Spectrum Imaging System demonstrates bioluminescence signal from McA-RH7777-CBRluc cells after addition of D-luciferin.

**Figure 4:**
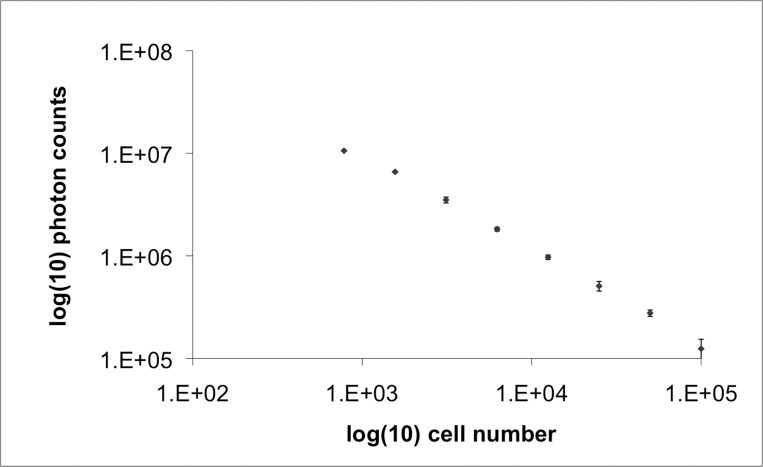
Quantitative relationship between cell concentration and bioluminescence signal in vitro. The plot shows a linear relationship between cell count in vitro and bioluminescence signal. Vertical bars represent standard deviation.

As shown in Figures 3-4, McA-RH7777-CBRluc cells harvested at eight passages after retroviral transfection demonstrate strong bioluminescence signal *in vitro* after the addition of D-luciferin, and a linear relationship exists between cell concentration and bioluminescence signal. Bioluminescence signal was tested at later passages and showed similar results (data not shown), confirming stable retroviral integration.

### Monitoring tumor growth in vivo with BLI

**Figure 5:**
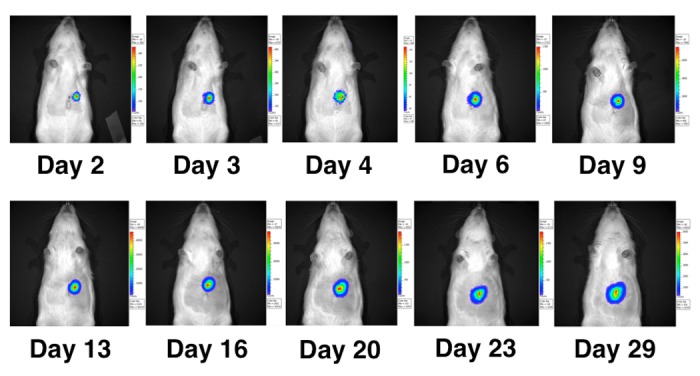
Serial measurements of BLI signal. Bioluminescence (p/sec/cm2/sr) was measured on days 2, 3, 4, 6, 9, 13, 16, 20, 23, and 29 in 5 animals.

**Figure 6:**
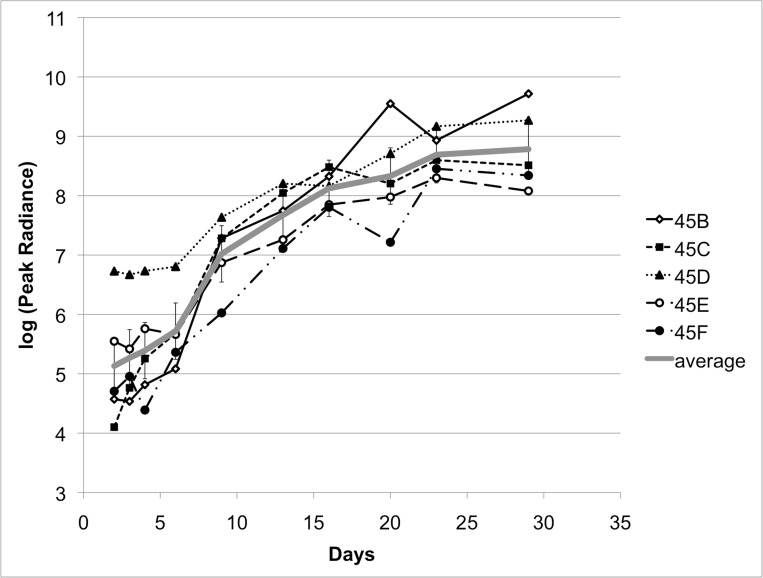
Quantitative values of serial measurements of BLI signal. Bioluminescence (p/sec/cm2/sr) was measured on days 2, 3, 4, 6, 9, 13, 16, 20, 23, and 29 in 5 animals. Mean values (circles) and ranges (vertical bars) are shown.

In the five animals that underwent serial bioluminescence imaging, tumor growth was first detected with BLI at Day 4 post-injection with a mean 2.9x10^4 p/sec/cm²/sr (range 4.7x10^3 – 1.5x10^5). The BLI signal increased linearly with the tumor size before reaching a plateau on Days 21-28 (Figures 5-6).

### Monitoring tumor growth with PET imaging and ultrasound

**Figure 7:**
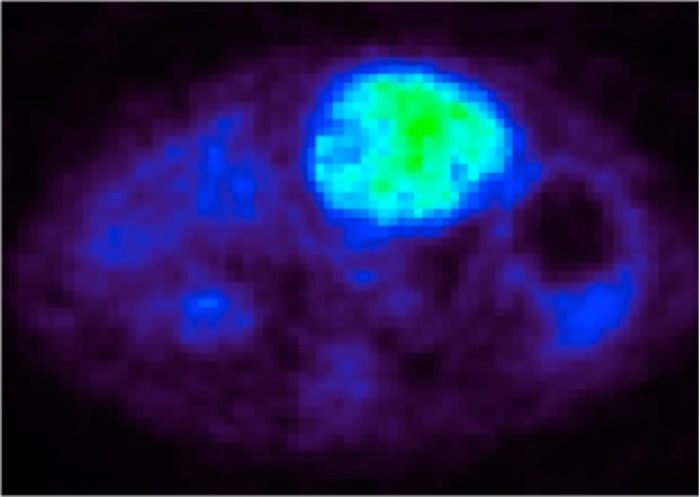
Serial measurements of PET signal. PET signal was measured on Days 7, 10, 15, 17, 21, 24, and 28 in 5 animals.

**Figure 8:**
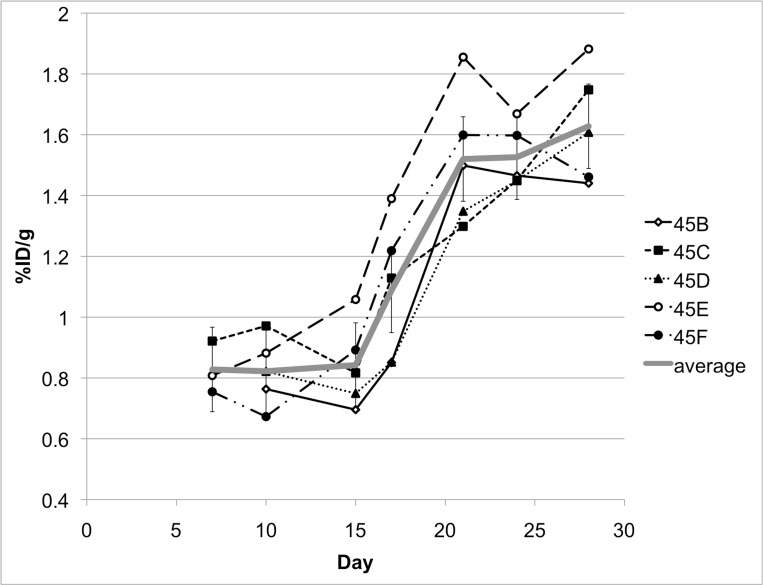
Quantitative serial measurements of PET signal. PET signal (%ID/g, triangle) was measured on Days 7, 10, 15, 17, 21, 24, and 28 in 5 animals. Mean values and ranges (vertical bars) are shown.

Serial PET imaging was performed at 10 time points. During PET image acquisition, the liver background signal remained constant with a mean tissue-specific uptake index of 0.04%ID/g (range 0.03-0.05). All six tumors demonstrated FDG uptake. A discrete tumor was first observed on Days 14-15 in all animals with a mean tissue-specific uptake index of 0.14%ID/g. The PET signal increased linearly with tumor growth with a maximum mean tissue-specific uptake index of 0.81%ID/g (range 0.59-0.91) on Day 28 (Figures 7-8). After Day 24, PET imaging revealed evidence of intraperitoneal tumor metastases in all animals, which was confirmed on necropsy.

In the 31 animals with middle lobe tumor implants, PET imaging was performed on Day 14. Three animals failed to demonstrate a hypermetabolic PET signal in the site of implantation; those animals were euthanized, and no tumor was seen on gross inspection. The mean tissue specific update index on Day 14 in the other 28 animals was 0.65%ID/g (range 0.35 to 0.87). The increased mean PET signal at Day 14 in these 31 animals, when compared with the original six animals, is attributable to the implantation of late log phase cells (70% confluence) in the initial six animals, which would lead to slower tumor growth, versus mid-log phase cells (50% confluence) in the subsequent 31 animals. Successful tumor implantation was achieved in 34 of 37 (91.9%) of the animals.

**Figure 9:**
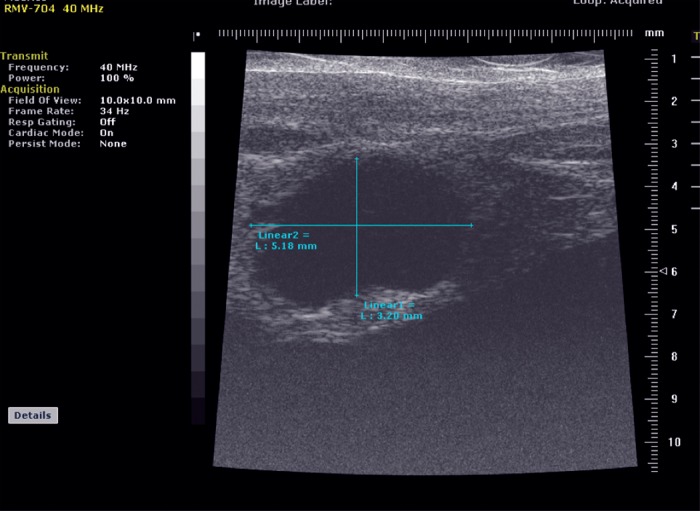
Representative image from ultrasound imaging on Day 17 from a single animal. Serial estimates of tumor volume (mm^3^) were calculated from high-frequency ultrasound measurements in three dimensions, taken on Days 3, 9, 14, 16, 20, 23, and 27.

**Figure 10:**
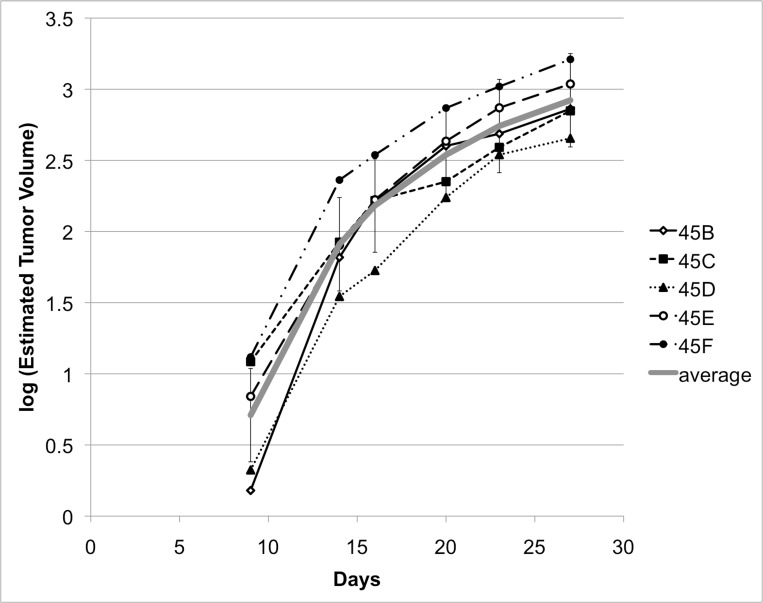
Quantitative representation of tumor volume from ultrasound. Serial estimates of tumor volume (mm^3^) were calculated from high-frequency ultrasound measurements in three dimensions, taken on Days 3, 9, 14, 16, 20, 23, and 27. Graph represents log of estimated volumes. Gray line represents average of log values; vertical bars represent standard error. No appreciable tumor was seen on Day 3 for all animals.

On Days 10-12, a discrete hypoechoic liver tumor was observed in all five serially imaged animals by high-frequency ultrasound with a mean maximum tumor diameter of 2.7 mm (range: 2.1-4.6). The tumors grew to a mean maximum of 11.3 mm (range 10.5-12.2) by Day 28 (Figures 9-10).

## Discussion

Driven by the need to study the pathophysiology and treatment of HCC, numerous groups have developed animal models of HCC that range in size from the mouse to the pig. Two basic strategies have been implemented in the creation of animal models of HCC: the generation of spontaneous tumors in the liver or the implantation of tumor cells into the animal.

Spontaneous tumor models are particularly useful in investigating the pathogenesis of HCC, as the lesions created by this technique go through stages of DNA damage and dysplasia as would be expected in the natural course of the disease. These models are also more likely to have tumors developing in a background of cirrhosis or fibrosis, mirroring what is present in the majority of patients with HCC. One technique for inducing HCC is the administration of the carcinogen diethylnitrosamine (DEN) with or without phenobarbital. After receiving DEN over several weeks either through diet or injections, the subjects develop multifocal HCC. This technique is most widely used in rats [22-29] but has also been described in rabbits [30] and in pigs [31]. Other methods to generate spontaneous HCC in rats include the administration of the carcinogen *p*-dimethylaminoazobenzene [32] or implementation of a methyl-deficient [33] or choline-deficient ethionine-supplemented diet [34].

Spontaneous HCC formation without chemical intervention has also been documented in a number of animal models. A strain of rat, the Long-Evans cinnamon rat, is predisposed to hereditary hepatitis with eventual development of HCC [35]. Various transgenic mice have demonstrated a high incidence of HCC [36-40]. A high incidence of HCC formation is also seen in the Eastern American woodchuck when infected with the woodchuck hepatitis virus, which is similar to the human hepatitis B virus [41-43].

A major disadvantage of spontaneous tumor models of HCC is the long incubation time prior to tumor formation, approximately 12-16 weeks for rat carcinogen models and several months for the rabbit and pig carcinogen models. This delay is not conducive to high-throughput studies. In addition, multiple tumors develop, and the growth trajectory of the tumors is variable. In the rat spontaneous HCC models, many of the study subjects died from the natural course of the disease prior to completion of the studies. This inherent variability in the time course of tumor development would limit the investigator’s ability to detect differences in the outcome after treatment.

Direct tumor implantation into animal subjects confers the advantages of rapid tumor development and control over tumor location. A facile method of tumor implantation is in the subcutaneous tissues of the animal [44-45]. However, while this technique may allow for assessment of the efficacy of intravenous therapies and direct intratumoral injections, intra-arterial delivery cannot be assessed, and the heterotopic localization may alter the tumor’s behavior compared with implantation in the liver.

Consequently, orthotopic implantation of tumor into the liver has been explored by various groups. Orthotopic mouse models of HCC have been created by xenotransplantation of human HCC cells into nude mice [46-51]. While this technique has the benefit of using human HCC cells, the behavior of the tumor and the therapies may be altered by the immunocompromised state of the host. Orthotopic implantation of murine HCC into immune-competent mice has also been described [52]. The small size of the mouse, however, allows for investigation of only systemic and, possibly, direct intratumoral administration of therapy, as the vessels are too small for intra-arterial delivery.

In contrast with the mouse, the vasculature of the rat is of adequate size to permit intra-arterial delivery of therapies, in addition to intratumoral and intravenous delivery. Orthotopic rat models of HCC have been developed in Buffalo rats using McA-RH7777 or ACI rats using Morris hepatoma 3924A [16-20]. The rat’s relatively small size allows for ease of handling, and the costs are substantially reduced when compared with larger animals, allowing for high-throughput studies. The small size is also advantageous in that tumors can be tracked longitudinally in a non-invasive fashion not only with PET imaging but also with BLI, which has a more limited penetration of tissue. This obviates the difficulties associated with generating and handling radiotracers.

In this paper, we describe a novel rat orthotopic HCC model that uses stably transduced tumor cells to allow longitudinal tracking with multiple non-invasive imaging modalities. While the implantation of Morris hepatoma cells has been described previously, our model is novel in the use of stable bioluminescence gene expression to allow tracking of tumor growth. The bioluminescence marker that we have selected — click beetle red luciferase — is notable in that it emits red light, which is attenuated less in deep tissues than light of shorter wavelengths [53-54].

This is also, to our knowledge, the first report demonstrating that implanted McA-RH7777 cells show a positive signal with [18]F-FDG PET imaging, providing a second means of tumor tracking. Of note, [18]F-FDG PET provides physiologic, as opposed to anatomic, imaging, and may be a better marker of tumor viability in the small animal than CT, MRI, or ultrasound. Likewise, as BLI signal can be generated only by live cells, it may also better track tumor viability than conventional anatomic imaging modalities. The ability to track change in tumor viability non-invasively is key for high-throughput evaluations of novel therapies, as it reduces the number of animals required to demonstrate statistical significance and avoids the errors inherent in assuming that all implanted animals must begin with the same tumor size when studying potential therapies. The ability to detect the tumor by ultrasound, as we found to be possible, allows the potential for percutaneous intratumoral delivery of therapies.

This model also has the advantage of rapid tumor development when compared with spontaneous models. All animals with tumor growth demonstrated PET signal in 14-15 days, and the six animals that underwent BLI demonstrated signal in four days.

A limitation of this model for intra-arterial delivery, when compared with larger animal models, is that it cannot be used for repeat intra-arterial delivery. Also, lobar delivery cannot be performed, as injection must be performed via a ligated gastroduodenal artery in order to cannulate a vessel of adequate size. In addition, unlike with spontaneous HCC models, the background liver parenchyma is non-cirrhotic, which differs from the majority of HCC patients. Lastly, it would be convenient if a serum marker for tumor growth were available. Although the ability to correlate tumor size with rat alpha-fetoprotein has been described [55], to date, we have not found a commercial test for alpha-fetoprotein that demonstrates cross-reactivity with rat alpha-fetoprotein to allow serum monitoring (data not shown).

## Conclusions

As novel pharmacologic, genetic, and combination therapies are being developed to treat HCC, investigators will need to be able to rapidly, and relatively inexpensively, test the therapies for safety and efficacy prior to large animal or human studies. With this model, high-throughput experiments can be performed using targeted routes of delivery — intra-arterial and intratumoral — in addition to intravenous delivery. The use of molecular imaging techniques in this small-animal model to track changes in tumor size provides a new platform with which investigators can advance the field of image-guided interventions in the treatment of HCC.

## References

[1] Llovet JM, Burroughs A, Bruix J (2003). Hepatocellular carcinoma. Lancet.

[2] Iwatsuki S, Starzl TE, Sheahan DG, Yokoyama I, Demetris AJ, Todo S, Tzakis AG, Van Thiel DH, Carr B, Selby R (1991). Hepatic resection versus transplantation for hepatocellular carcinoma. Ann Surg.

[3] Kalva SP, Thabet A, Wicky S (2008). Recent advances in transarterial therapy of primary and secondary liver malignancies. Radiographics.

[4] Llovet JM, Ricci S, Mazzaferro V, Hilgard P, Gane E, Blanc JF, de Oliveira AC, Santoro A, Raoul JL, Forner A, Schwartz M, Porta C, Zeuzem S, Bolondi L, Greten TF, Galle PR, Seitz JF, Borbath I, Häussinger D, Giannaris T, Shan M, Moscovici M, Voliotis D, Bruix J, SHARP Investigators Study Group (2008). Sorafenib in advanced hepatocellular carcinoma. NEJM.

[5] Kim YI, Chung JW, Park JH, Han JK, Hong JW, Chung H (2006). Intraarterial gene delivery in rabbit hepatic tumors: transfection with nonviral vector by using iodized oil emulsion. Radiology.

[6] Nakakuma K, Tashiro S, Hiraoka T, Ogata K, Ootsuka K (1985). Hepatocellular carcinoma and metastatic cancer detected by iodized oil. Radiology.

[7] Yamashita Y, Takahashi M, Fukushima S, Nishida M, Nakano M (1987). Experimental study of hepatic artery embolization: evaluation of various embolic materials. Radiat Med.

[8] Yumoto Y, Jinno K, Inatsuki S, Moriwaki S, Hanafusa T, Yumoto E, Shiota T, Higashi T, Koide N, Hada H (1992). Treatment of hepatocellular carcinoma by transcatheter hepatic arterial injection of radioactive iodized oil solution. Cancer Chemother Pharmacol.

[9] Ko YH, Pedersen PL, Geschwind JF (2001). Glucose catabolism in the rabbit VX2 tumor model for liver cancer: characterization and targeting hexokinase. Cancer Lett.

[10] Yoon CJ, Chung JW, Park JH, Yoon YH, Lee JW, Jeong SY, Chung H (2003). Transcatheter arterial chemoembolization with paclitaxel-lipiodol solution in rabbit VX2 liver tumor. Radiology.

[11] Rhee TK, Young JY, Larson AC, Haines GK 3rd, Sato KT, Salem R, Mulcahy MF, Kulik LM, Paunesku T, Woloschak GE, Omary RA (2007). Effect of transcatheter arterial embolization on levels of hypoxia-inducible factor-1alpha in rabbit VX2 liver tumors. J Vasc Interv Radiol.

[12] Shah SS, Jacobs DL, Krasinkas AM, Furth EE, Itkin M, Clark TW (2004). Percutaneous ablation of VX2 carcinoma-induced liver tumors with use of ethanol versus acetic acid: pilot study in a rabbit model. J Vasc Interv Radiol.

[13] Hong K, Kobeiter H, Georgiades CS, Torbenson MS, Geschwind JF (2005). Effects of the type of embolization particles on carboplatin concentration in liver tumors after transcatheter arterial chemoembolization in a rabbit model of liver cancer. J Vasc Interv Radiol.

[14] Lee KH, Liapi E, Ventura VP, Buijs M, Vossen JA, Vali M, Geschwind JF (2008). Evaluation of different calibrated spherical polyvinyl alcohol microspheres in transcatheter arterial chemoembolization: VX2 tumor model in rabbit liver. J Vasc Interv Radiol.

[15] Cao W, Wan Y, Liang ZH, Duan YY, Liu X, Wang ZM, Liu YY, Zhu J, Liu XT, Zhang HX (2010). Heated lipiodol as an embolization agent for transhepatic arterial embolization in VX2 rabbit liver cancer model. Eur J Radiol.

[16] Maataoui A, Qian J, Vossoughi D, Khan MF, Oppermann E, Bechstein WO, Vogl TJ (2005). Transarterial chemoembolization alone and in combination with other therapies: a comparative study in an animal HCC model. Eur Radiol.

[17] Trübenbach J, Graepler F, Pereira PL, Ruck P, Lauer U, Gregor M, Claussen CD, Huppert PE (2000). Growth characteristics and imaging properties of the morris hepatoma 3924A in ACI rats: a suitable model for transarterial chemoembolization. Cardiovasc Intervent Radiol.

[18] Altomonte J, Braren R, Schulz S, Marozin S, Rummeny EJ, Schmid RM, Ebert O (2008). Synergistic antitumor effects of transarterial viroembolization for multifocal hepatocellular carcinoma in rats. Hepatology.

[19] Barajas M, Mazzolini G, Genové G, Bilbao R, Narvaiza I, Schmitz V, Sangro B, Melero I, Qian C, Prieto J (2001). Gene therapy of orthotopic hepatocellular carcinoma in rats using adenovirus coding for interleukin 12. Hepatology.

[20] Ebert O, Shinozaki K, Huang TG, Savontaus MJ, Garcia-Sastre A, Woo SL (2003). Oncolytic vesicular stomatitis virus for treatment of orthotopic hepatocellular carcinoma in immune-competent rats. Cancer Res.

[21] Swift S, Lorens J, Achacoso P, Nolan GP (2001). Rapid production of retroviruses for efficient gene delivery to mammalian cells using 293T cell-based systems. Curr Protoc Immunol.

[22] Michoux N, Huwart L, Abarca-Quinones J, Dorvillius M, Annet L, Peeters F, Van Beers BE (2008). Transvascular and interstitial transport in rat hepatocellular carcinomas: dynamic contrast-enhanced MRI assessment with low- and high-molecular weight agents. J Magn Reson Imaging.

[23] Solt DB, Medline A, Farber E (1977). Rapid emergence of carcinogen-induced hyperplastic lesions in a new model for the sequential analysis of liver carcinogenesis. Am J Pathol.

[24] Mathis JM, Williams BJ, Sibley DA, Carroll JL, Li J, Odaka Y, Barlow S, Nathan CO, Li BD, DeBenedetti A (2006). Cancer-specific targeting of an adenovirus-delivered herpes simplex virus thymidine kinase suicide gene using translational control. J Gene Med.

[25] Kwon HC, Kim JH, Kim KC, Lee KH, Lee JH, Lee BH, Lee KH, Jang JJ, Lee CT, Lee H, Kim CM (2001). In vivo antitumor effect of herpes simplex virus thymidine kinase gene therapy in rat hepatocellular carcinoma: feasibility of adenovirus-mediated intra-arterial gene delivery. Mol Cells.

[26] Shiba H, Okamoto T, Futagawa Y, Misawa T, Yanaga K, Ohashi T, Eto Y (2006). Adenovirus vector-mediated gene transfer using degradable starch microspheres for hepatocellular carcinoma in rats. J Surg Res.

[27] Fournier LS, Cuenod CA, de Bazelaire C, Siauve N, Rosty C, Tran PL, Frija G, Clement O (2004). Early modifications of hepatic perfusion measured by functional CT in a rat model of hepatocellular carcinoma using a blood pool contrast agent. Eur Radiol.

[28] Tada M, Hatano E, Taura K, Nitta T, Koizumi N, Ikai I, Shimahara Y (2006). High volume hydrodynamic injection of plasmid DNA via the hepatic artery results in a high level of gene expression in rat hepatocellular carcinoma induced by diethylnitrosamine. J Gene Med.

[29] Kuroiwa-Trzmielina J, de Conti A, Scolastici C, Pereira D, Horst MA, Purgatto E, Ong TP, Moreno FS (2009). Chemoprevention of rat hepatocarcinogenesis with histone deacetylase inhibitors: efficacy of tributyrin, a butyric acid prodrug. Int J Cancer.

[30] Reznik GK, Padberg G (1991). Diethylnitrosamine-induced metastasizing hepatocellular carcinomas in New Zealand white rabbits. A tumor model for clinical investigations. J Cancer Res Clin Oncol.

[31] Li X, Zhou X, Guan Y, Wang YX, Scutt D, Gong QY (2006). N-nitrosodiethylamine-induced pig liver hepatocellular carcinoma model: radiological and histopathological studies. Cardiovasc Intervent Radiol.

[32] Murugan RS, Uchida K, Hara Y, Nagini S (2008). Black tea polyphenols modulate xenobiotic-metabolizing enzymes, oxidative stress and adduct formation in a rat hepatocarcinogenesis model. Free Radic Res.

[33] Bagnyukova TV, Tryndyak VP, Muskhelishvili L, Ross SA, Beland FA, Pogribny IP (2008). Epigenetic downregulation of the suppressor of cytokine signaling 1 (Socs1) gene is associated with the STAT3 activation and development of hepatocellular carcinoma induced by methyl-deficiency in rats. Cell Cycle.

[34] Kubota K, Soeda J, Misawa R, Mihara M, Miwa S, Ise H, Takahashi M, Miyagawa S (2008). Bone marrow-derived cells fuse with hepatic oval cells but are not involved in hepatic tumorigenesis in the choline-deficient ethionine-supplemented diet rat model. Carcinogenesis.

[35] Nakakoshi T, Kajiyama M, Fujita N, Jong-Hon K, Takeichi N, Miyasaka K (1995). Magnetic resonance imaging of Long-Evans cinnamon rats as a new model of hepatocellular carcinoma. Acad Radiol.

[36] Takai A, Toyoshima T, Uemura M, Kitawaki Y, Marusawa H, Hiai H, Yamada S, Okazaki IM, Honjo T, Chiba T, Kinoshita K (2009). A novel mouse model of hepatocarcinogenesis triggered by AID causing deleterious p53 mutations. Oncogene.

[37] von Falck C, Rodt T, Halter R, Spanel R, Galanski M, Borlak J (2009). Combined microPET/CT for imaging of hepatocellular carcinoma in mice. Front Biosci (Landmark Ed).

[38] Yen CH, Hung JH, Ueng YF, Liu SP, Chen SY, Liu HH, Chou TY, Tsai TF, Darbha R, Hsieh LL, Chen YM (2009). Glycine N-methyltransferase affects the metabolism of aflatoxin B1 and blocks its carcinogenic effect. Toxicol Appl Pharmacol.

[39] Lakhtakia R, Kumar V, Reddi H, Mathur M, Dattagupta S, Panda SK (2003). Hepatocellular carcinoma in a hepatitis B 'x' transgenic mouse model: A sequential pathological evaluation. J Gastroenterol Hepatol.

[40] Sun Q, Zhang Y, Liu F, Zhao X, Yang X (2007). Identification of candidate biomarkers for hepatocellular carcinoma through pre-cancerous expression analysis in an HBx transgenic mouse. Cancer Biol Ther.

[41] Gouillat C, Manganas D, Zoulim F, Vitrey D, Saguier G, Guillaud M, Ain JF, Duque-Campos R, Jamard C, Praves M, Trepo C (1997). Woodchuck hepatitis virus-induced carcinoma as a relevant natural model for therapy of human hepatoma. J Hepatol.

[42] Bilbao R, Gérolami R, Bralet MP, Qian C, Tran PL, Tennant B, Prieto J, Bréchot C (2000). Transduction efficacy, antitumoral effect, and toxicity of adenovirus-mediated herpes simplex virus thymidine kinase/ ganciclovir therapy of hepatocellular carcinoma: the woodchuck animal model. Cancer Gene Ther.

[43] Tennant BC, Toshkov IA, Peek SF, Jacob JR, Menne S, Hornbuckle WE, Schinazi RD, Korba BE, Cote PJ, Gerin JL (2004). Hepatocellular carcinoma in the woodchuck model of hepatitis B virus infection. Gastroenterology.

[44] Ain JF, Gouillat C, Bertrand S, Fourel I, Guillaud M, Saguier G, Trepo C (1994). Human hepatocellular carcinoma transplanted in nude mice: a relevant experimental model to assess tumoral destruction by alcoholization. J Surg Res.

[45] Iida T, Shiba H, Misawa T, Ohashi T, Eto Y, Yanaga K (2008). Adenovirus-mediated CD40L gene therapy induced both humoral and cellular immunity against rat model of hepatocellular carcinoma. Cancer Sci.

[46] Ding L, Chen XP, Zhang ZW, Jing K, Zhang WG (2007). Human multi-drug resistant hepatocellular carcinoma induced in nude mice by B-ultrasonographically-directed orthotopic implantation: a new experimental model. Hepatobiliary Pancreat Dis Int.

[47] Schmitz V, Tirado-Ledo L, Tiemann K, Raskopf E, Heinicke T, Ziske C, González-Carmona MA, Rabe C, Wernert N, Prieto J, Qian C, Sauerbruch T, Caselmann WH (2004). Establishment of an orthotopic tumour model for hepatocellular carcinoma and non-invasive in vivo tumour imaging by high resolution ultrasound in mice. J Hepatol.

[48] Yang BW, Liang Y, Xia JL, Sun HC, Wang L, Zhang JB, Tang ZY, Liu KD, Chen J, Xue Q, Chen J, Gao DM, Wu WZ (2008). Biological characteristics of fluorescent protein-expressing human hepatocellular carcinoma xenograft model in nude mice. Eur J Gastroenterol Hepatol.

[49] Wang Y, Sun Z, Peng J, Zhan L (2007). Bioluminescent imaging of hepatocellular carcinoma in live mice. Biotechnol Lett.

[50] Yao X, Hu JF, Daniels M, Yien H, Lu H, Sharan H, Zhou X, Zeng Z, Li T, Yang Y, Hoffman AR (2003). A novel orthotopic tumor model to study growth factors and oncogenes in hepatocarcinogenesis. Clin Cancer Res.

[51] Gao YS, Chen XP, Li KY, Wu ZD (2004). Nude mice model of human hepatocellular carcinoma via orthotopic implantation of histologically intact tissue. World J Gastroenterol.

[52] Hsieh JL, Lee CH, Teo ML, Lin YJ, Huang YS, Wu CL, Shiau AL (2009). Transthyretin-driven oncolytic adenovirus suppresses tumor growth in orthotopic and ascites models of hepatocellular carcinoma. Cancer Sci.

[53] Zhao H, Doyle TC, Coquoz O, Kalish F, Rice BW, Contag CH (2005). Emission spectra of bioluminescent reporters and interaction with mammalian tissue determine the sensitivity of detection in vivo. J Biomed Opt.

[54] Miloud T, Henrich C, Hämmerling GJ (2007). Quantitative comparison of click beetle and firefly luciferases for in vivo bioluminescence imaging. J Biomed Opt.

[55] Sell S (1974). The catabolism of alpha1-fetoprotein and albumin in rats bearing Morris hepatoma 7777. Cancer Res.

